# Deciphering the role of KLRB1: a novel prognostic indicator in hepatocellular carcinoma

**DOI:** 10.1186/s12876-024-03299-4

**Published:** 2024-06-24

**Authors:** Siting Fang, Yinglu Zhou

**Affiliations:** 1 Department of Stomatology, Huashan hospital, Fudan university, Shanghai, 200040 China; 2 Nursing Department, Huashan Hospital Fudan University, Shanghai, 200040 China

**Keywords:** KLRB1, HCC, Recurrence, T cell, NK cell

## Abstract

**Background:**

Hepatocellular carcinoma (HCC) represents a significant global health challenge with high incidence and mortality rates. T cells and natural killer (NK) cells are pivotal in this context, yet HCC can evade immune surveillance. CD161 (KLRB1), a C-type lectin receptor, modulates immune responses and is expressed on NK cells and a subset of T cells. Its relevance in HCC remains poorly understood, with conflicting findings regarding its impact on patient prognosis.

**Methods:**

Utilizing TCGA data and single-cell analysis, we investigated the biological functions of KLRB1 in HCC. Peripheral blood samples from 126 HCC patients were collected to assess KLRB1 expression on NK and T cells. The diagnostic performance of KLRB1 on NK and CD8 + T cells was evaluated using receiver operating characteristic curve (ROC) analysis, while its prognostic significance was assessed using Kaplan-Meier analysis and COX regression models.

**Results:**

Analysis of TCGA data revealed a significant correlation between KLRB1 expression and immune activation, particularly T cell activation. Single-cell data further demonstrated elevated KLRB1 expression in tissue-resident NK and T cells within HCC, which co-expressed markers of immune activation. Clinical data showed downregulated KLRB1 expression on NK and T cells in HCC patients compared to health individuals, with lower expression levels correlating with poorer prognosis.

**Conclusion:**

KLRB1 emerges as a promising biomarker in HCC, with its downregulation on peripheral blood NK and T cells suggesting potential prognostic value. Further elucidation of KLRB1’s role in HCC may pave the way for the development of targeted immunotherapies and the improvement of patient outcomes.

**Supplementary Information:**

The online version contains supplementary material available at 10.1186/s12876-024-03299-4.

## Introduction

Hepatocellular carcinoma poses a grave health concern in contemporary society, exhibiting notably high incidence and mortality rates worldwide. Despite ranking sixth among the most prevalent malignancies globally, it claims the third position in terms of mortality rates, trailing only behind lung and gastric cancers, highlighting its lethal nature [1]. While therapeutic approaches and medications for hepatocellular carcinoma have shown gradual progress and development in recent years, the recurrence rate remains stubbornly high. Even accepting proper treatment, approximately 20% of patients experience either local recurrence or distant metastasis [2, 3]. Alpha-fetoprotein (AFP), a classic hepatocellular carcinoma-specific diagnostic and prognostic marker, still presents limitations. Some patients face recurrence and metastasis despite having negative AFP levels. Consequently, identifying suitable prognostic markers for early HCC screening remains the most pressing clinical challenge.

The tumor microenvironment is a crucial factor influencing the onset and progression of tumors [4, 5]. It consists of various infiltrating cells, including immune cells, endothelial cells, fibroblasts, and a diverse array of extracellular matrix components such as cytokines and chemokines, collectively shaping a complex tumor microecological environment. Studies have highlighted the significance of T cells and NK cells within tumor tissues as pivotal in inhibiting tumor growth [6–11]. CD8^+^ T cells and NK cells are capable of directly identifying and eliminating tumors, while CD4^+^ T cells contribute indirectly by producing cytokines like interferon-gamma(IFN-γ) and tumor necrosis factor-alpha(TNF-a), thus participating in anti-tumor immune responses. However, in the context of HCC, tumor cells can employ various strategies to evade immune attacks, including suppressing T cell activity, diminishing NK cell function, and inducing an increase in immunosuppressive cells [12, 13]. Consequently, this promotes tumor growth and metastasis. Hence, investigating the roles and interplay of T cells and NK cells within the HCC tumor microenvironment holds promise in developing novel molecular markers and immunotherapy strategies aimed at enhancing treatment outcomes for patients with HCC.

KLRB1(CD161), also referred to as Natural Killer Cell Receptor 1 A(NKR-P1A), is a cell surface molecule. It falls under the category of C-type lectin receptors and is predominantly expressed on the surfaces of natural killer cells and a subset of T cells. The functionality of KLRB1 involves regulating immune responses, including modulation of NK cell and T cell activity, as well as participation in immune reactions. It is believed to play a significant role in antiviral immunity and is associated with the development of autoimmune diseases, infectious diseases, and cancers [14]. In recent years, research on KLRB1 in tumors has gained significant attention. However, the findings from these studies are diverse. While some suggest a correlation between high KLRB1 expression in tumors and poorer patient prognosis, others propose that KLRB1 may enhance the anti-tumor capabilities of immune cells, potentially improving patient outcomes [15–18]. Research specifically focusing on the association of KLRB1 with HCC is relatively scarce. Therefore, this study aims to systematically investigate the role of KLRB1 in HCC, evaluating its potential correlation with clinical prognosis inHCC patients and assessing its clinical significance.

## Materials and methods

### Enrollment and specimen collection

In this study, a total of 126 patients diagnosed with hepatocellular carcinoma were enrolled from July 2017 to October 2018. All patients met the diagnostic criteria outlined in the Guidelines for the Diagnosis and Treatment of Hepatocellular Carcinoma (2019 Edition) and were undergoing their initial diagnosis, followed by surgical resection treatment at our hospital. Exclusion criteria included: (1) presence of other major diseases such as severe cardiovascular or autoimmune diseases; (2) prior treatment at other medical facilities; (3) history of other tumor diseases. Additionally, 50 healthy individuals were recruited as controls. Ethical approval for this study was obtained from the Ethics Committee of Huashan Hospital, and informed consent was obtained from all participants, including patients and healthy controls. The clinicopathologic characteristics of HCC patients and healthy individuals enrolled in the research was shown in Table 1.

**Table 1:** The clinicopathologic characteristics of HCC patients and healthy individuals enrolled in the research

Clinical Characteristic	Patients	Heathy Control
Age		
≤ 50	41	15
> 50	85	35
Sex		
Female	25	10
Male	101	40
HBsAg		
Negative	15	
Positive	111	
AFP		
≤ 400ng/mL	70	
> 400ng/mL	56	
Tumor number		
Single	87	
Multiple	39	
Tumor Size		
≤ 5 cm	88	
> 5 cm	38	
Tumor encapsulation		
None	86	
Complete	40	
Satellite lesions		
No	101	
Yes	25	
Vascular invasion		
No	77	
Yes	49	
Tumor grade		
I-II	83	
III-IV	43	
Child-Pugh grade		
A	120	
B	6	
BCLC stage		
0 + A	99	
B + C	27	
Recurrence		
No	79	
Yes	47	
Death		
No	88	
Yes	38	

2mL of EDTA anticoagulated blood from both preoperative HCC patients and individuals in the healthy control group was collected.

### The follow-up of HCC patients

The follow-up period begins upon the patient’s discharge from treatment and ends at the time of patient death or the last follow-up visit. Two months after receiving surgical treatment, patients undergo follow-up examinations, including serum blood tests (α-fetoprotein, alanine aminotransferase, etc.) and imaging tests. Subsequent follow-up examinations are arranged by physicians based on the patient’s individual condition. Time to recurrence (TTR) and overall survival (OS) are defined as the interval between surgery and recurrence or death.

### DATA Acquisition

The clinical information, HCC tissue RNA-sequence and fellow-up data of 371 HCC patients were obtained from The Cancer Genome Atlas (TCGA) (https://portal.gdc.cancer.gov). Single-cell RNA sequencing data of HCC tissue (GSE210679) was obtained from NCBI Geo DataSets(https://www.ncbi.nlm.nih.gov/gds/).

### Functional enrichment analysis

Utilizing the TCGA database, genes most strongly associated with KLRB1 were submitted to the Database for Annotation, Visualization, and Integrated Discovery (DAVID) for Gene Ontology (GO) and Kyoto Encyclopedia of Genes and Genomes (KEGG) analysis. Following this, the top 8 analysis results were showcased in the figure, prioritized by their P-values.

### Single-cell sequencing analysis

We utilized the R package (Seurat 4.3.0, dplyr 1.1.0, patchwork 1.1.2) to conduct single-cell sequencing data analysis, encompassing data normalization, principal component analysis (PCA), dimensionality reduction, T-distributed stochastic neighbor embedding (Tsne) clustering, and identification of differential expression.

### Peripheral blood mononuclear cells (PBMCs) isolation and flow cytometry detection

PBMCs, anticoagulated with 2mL of EDTA, were isolated using Ficoll (GE Healthcare) density gradient centrifugation. The intermediate white layer was carefully collected, followed by two washes with PBS. Cells were then resuspended in 200uL of PBS and set aside. Flow cytometry antibodies, including CD3-FITC, CD56/CD16-PE, CD45-Percp-Cy5.5, CD4-PE-Cy7, CD19-APC, CD8-APC-Cy7, and KLRB1-BV421 (BD Bioscience), were added as per the manufacturer’s instructions. After a 15-minute incubation in the dark, cells were washed once with PBS. Finally, cells were resuspended in 200uL of PBS for analysis using a flow cytometer (Canto II, BD Bioscience). The gating strategy was shown in Supplementary Fig. 2.

### Statistical analysis

This study utilized SPSS 19.0 and GraphPad Prism 6 for statistical analysis. Survival analyses were conducted using log-rank tests and presented in Kaplan-Meier graphs. Group differences were assessed using appropriate statistical tests, including two-tailed unpaired Student’s t-tests, Pearson’s χ2 tests, Mann-Whitney U tests, two-way ANOVA, or log-rank tests. Statistical significance was set at a two-tailed P-value < 0.05.

## Result

### KLRB1 expression and prognosis in HCC: insights from analysis of TCGA database

**Fig. 1:**
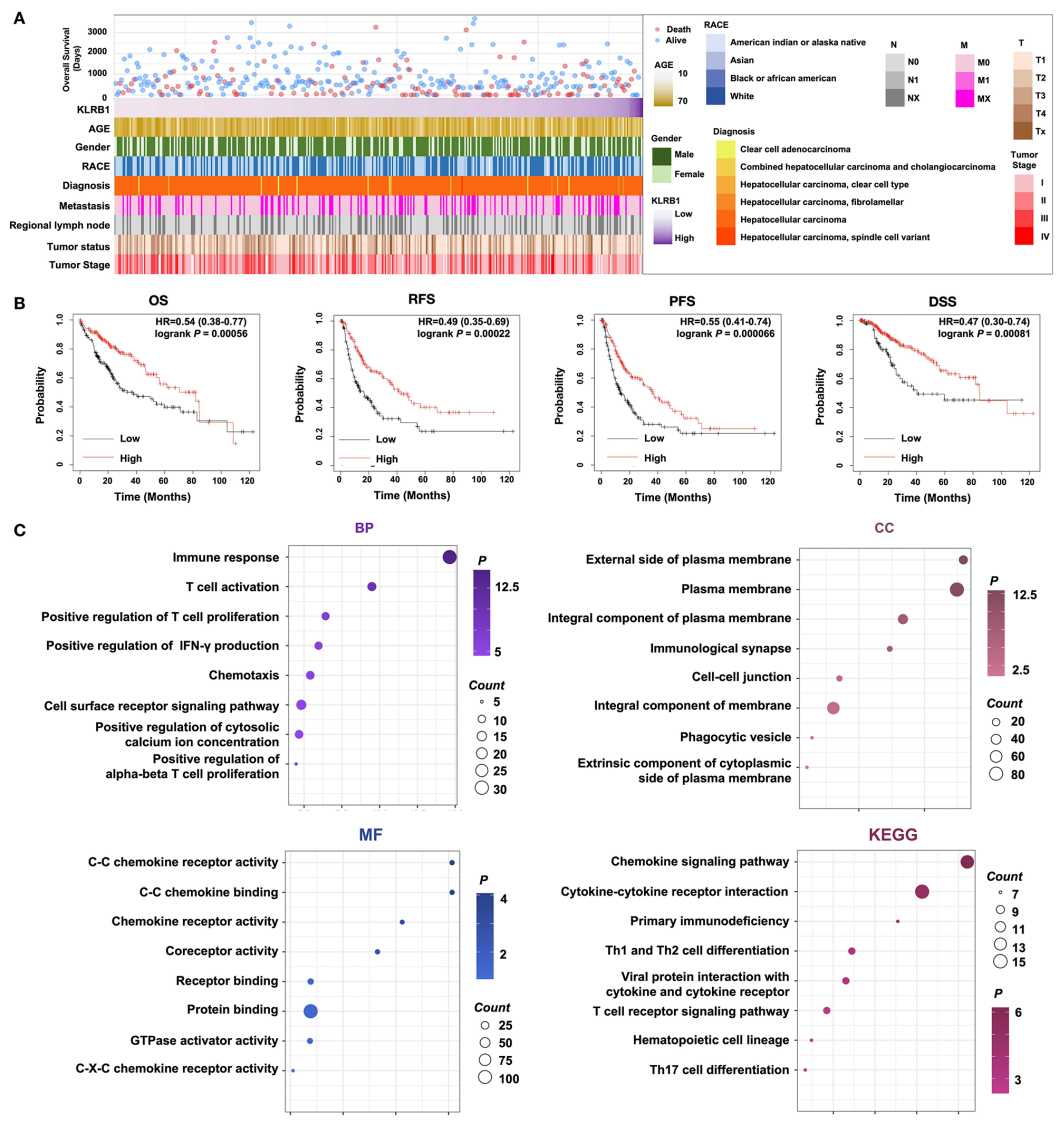
Correlation of KLRB1 with clinical parameters and prognosis of hepatocellular carcinoma patients in TCGA Database, and GO and KEGG analysis of KLRB1. **A**. The landscape of KLRB1-related clinicopathological information of HCC in the TCGA database. **B**. Kaplan‐Meier analysis of KLRB1 expression in the TCGA. **C**. Biological processes (BP), cellular components (CC), and molecular functions (MF) are mostly associated to KLRB1 in HCC

We gathered clinical information and tumor tissue RNA-seq data from 371HCC patients in the TCGA database. Our analysis revealed associations between KLRB1 expression levels and patient age, tumor status, and tumor staging. Notably, higher tumor staging correlated with lower KLRB1 expression (Fig. 1A and sFigure 1).

Using KLRB1 as a prognostic marker, we predicted the survival outcomes of the 371HCC patients. Results demonstrated that patients with high KLRB1 expression in tumor tissue exhibited significantly better prognoses compared to those with low KLRB1 expression. This contrast was evident across overall survival rate, progression-free survival rate, recurrence-free survival period, and disease-specific survival period metrics (Fig. 1B).

To delve deeper into the biological role of KLRB1 inHCC, we conducted Pearson analysis to identify genes correlated with KLRB1 expression, followed by GO and KEGG analyses. Our findings highlighted immune response and T cell activation as the predominant biological processes (BP) associated with KLRB1. Additionally, the cellular component (CC) most linked to KLRB1 was the external side of plasma membrane, while the primary molecular function (MF) was C-C chemokine receptor activity. Furthermore, KEGG analysis underscored the close relationship between KLRB1 and the Chemokine signaling pathway as well as Cytokine-cytokine receptor interaction (Fig. 1C).

### KLRB1 expression patterns in hepatocellular carcinoma tissues

**Fig. 2:**
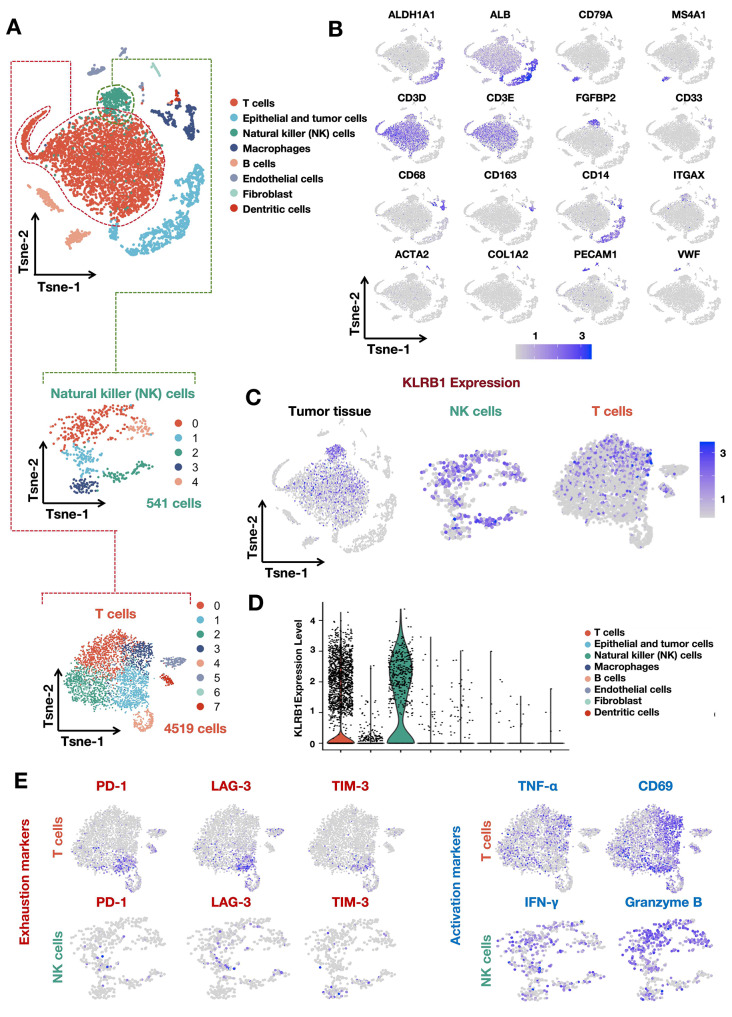
Expression patterns and cellular distribution of KLRB1 in hepatocellular carcinoma tissues analyzed by Single-Cell Sequencing. **A**. Standardized single-cell sequencing data of HCC tissues were subjected to dimensionality reduction clustering using PCA and t-SNE. **B**. Cell populations were annotated and distinguished using specific markers. **C-D**. KLRB1 primarily expressed on NK cells and T cells in HCC tissues, with higher levels in NK cells compared to T cells. E. Exhausted T cells and NK cells showed lower KLRB1 expression, while activated NK cells and T cells exhibited higher KLRB1 expression

In previous studies, the focus on KLRB1 has primarily centered around immune cells. However, the expression of KLRB1 in HCC tissues and its distribution within various cell types remain unclear. Therefore, we analyzed single-cell sequencing data of HCC tissues to further investigate the expression patterns of KLRB1 within tumor tissues. Initially, we standardized the single-cell sequencing data and then performed dimensionality reduction clustering using PCA and t-SNE. We annotated and distinguished different cell populations using specific markers (ALDH1A1 and ALB for epithelial cells and tumor cells, CD79A and MS4A1 for B cells, CD3D and CD3E for T cells, FGFBP2 for NK cells, CD33 for granulocytes, CD68, CD163, and CD14 for macrophages, ITGAX for dendritic cells, ACTA2 and COL1A2 for fibroblasts, PECAM1 and vWF for endothelial cells) (Fig. 2A-B) [19]. The results indicated that in HCC tissues, KLRB1 is primarily expressed on NK cells and T cells, with higher expression observed on NK cells compared to T cells (Fig. 2C-D). Furthermore, we annotated the activation and exhaustion states of cells (PD-1, LAG-3, and TIM-3 for exhaustion markers in NK cells and T cells; CD69 and TNF-α for activation markers in T cells; IFN-gamma and Granzyme B for activation markers in NK cells) and found that exhausted T cells and NK cells exhibited lower expression of KLRB1, whereas activated NK cells and T cells showed higher KLRB1 expression (Fig. 2E).

### The expression of KLRB1 in HCC patients with different outcome

**Fig. 3:**
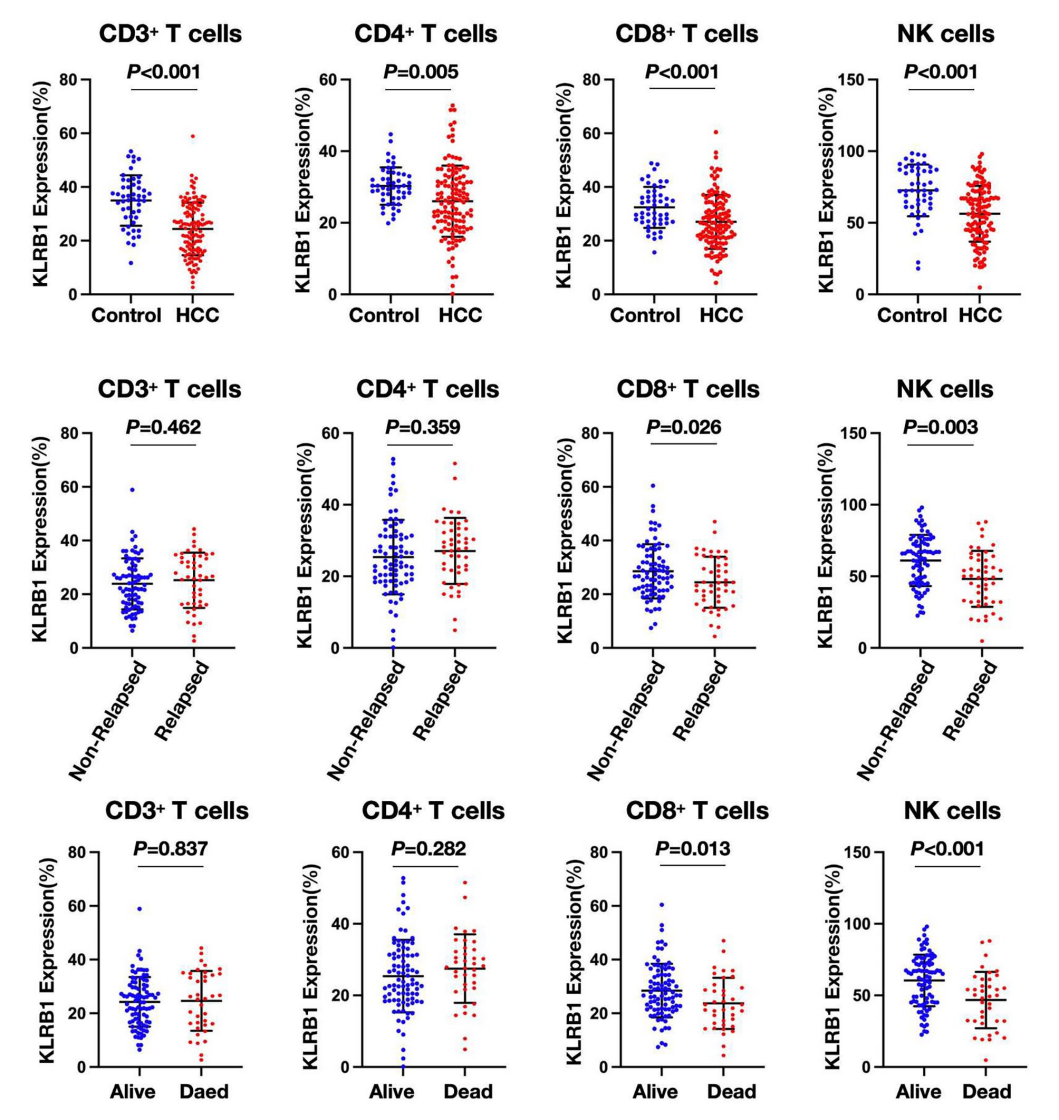
Decreased KLRB1 expression on CD3^+^ T cells, CD4^+^, CD8^+^ T cells, and NK cells in HCC patients compared to controls. Reduced KLRB1 expression correlates with adverse prognoses in HCC patients

Based on our comprehensive analysis of single-cell sequencing data, we have discerned that KLRB1 exhibits predominant expression on NK cells and T cells within hepatic tissue, with a noteworthy correlation observed between infiltrating immune cells in the tissue and those circulating peripherally [20]. Motivated by these findings, our study aims to elucidate whether the surface expression of KLRB1 on NK cells and T cells in the peripheral circulation of HCC patients could serve as a prognostic indicator. To this end, we meticulously collected peripheral blood samples from 126 HCC patients at their initial presentation to our hospital, alongside a meticulously matched cohort of 50 healthy controls. Peripheral blood samples were meticulously obtained prior to surgical intervention from the HCC patients, and the expression of KLRB1 on NK cells and T cells within the enrolled cohort was meticulously assessed utilizing flow cytometry techniques. Our meticulous analysis reveals that, relative to the healthy control cohort, HCC patients manifest decreased expression of KLRB1 on CD3^+^ T cells (total T cells) in their peripheral blood, as well as attenuated expression on CD4^+^ and CD8^+^ T cells. Furthermore, KLRB1 expression on NK cells among HCC patients is also notably diminished compared to the healthy counterpart. Moreover, through meticulous comparison of KLRB1 expression levels on T cells and NK cells among HCC patients with divergent prognostic outcomes, our findings underscore a significant reduction in KLRB1 expression on CD8^+^ T and NK cells among patients experiencing recurrence or mortality, in stark contrast to those with favorable prognoses (Fig. 3; Table 2). Collectively, this results suggest the potential utility of KLRB1 expression on CD8^+^ T and NK cells as a prognostic biomarker in HCC.

**Table 2:** Expression of KLRB1 in Peripheral Blood Immune Cells of Healthy Individuals and Liver Cancer Patients with Diverse Prognostic Status

	Healthy Control	HCC Patients	Relapsed	No-Relapsed	Alive	Dead
Number	50	126	79	47	88	38
CD3^+^ T cells (KLRB1%)	34.96 ± 9.38	24.37 ± 9.30	23.87 ± 9.51	25.20 ± 10.32	24.25 ± 9.23	24.64 ± 11.14
CD4^+^ T cells (KLRB1%)	30.26 ± 5.22	26.02 ± 9.97	25.39 ± 10.40	27.08 ± 9.21	25.39 ± 10.12	27.48 ± 9.57
CD8^+^ T cells (KLRB1%)	32.40 ± 7.61	27.02 ± 10.03	28.54 ± 10.07	24.45 ± 9.51	28.46 ± 9.95	23.68 ± 9.52
NK cells (KLRB1%)	72.65 ± 18.07	56.29 ± 19.47	61.09 ± 17.90	48.23 ± 19.52	60.40 ± 17.99	46.78 ± 19.65

### Prognostic significance of KLRB1 in HCC patients

**Fig. 4:**
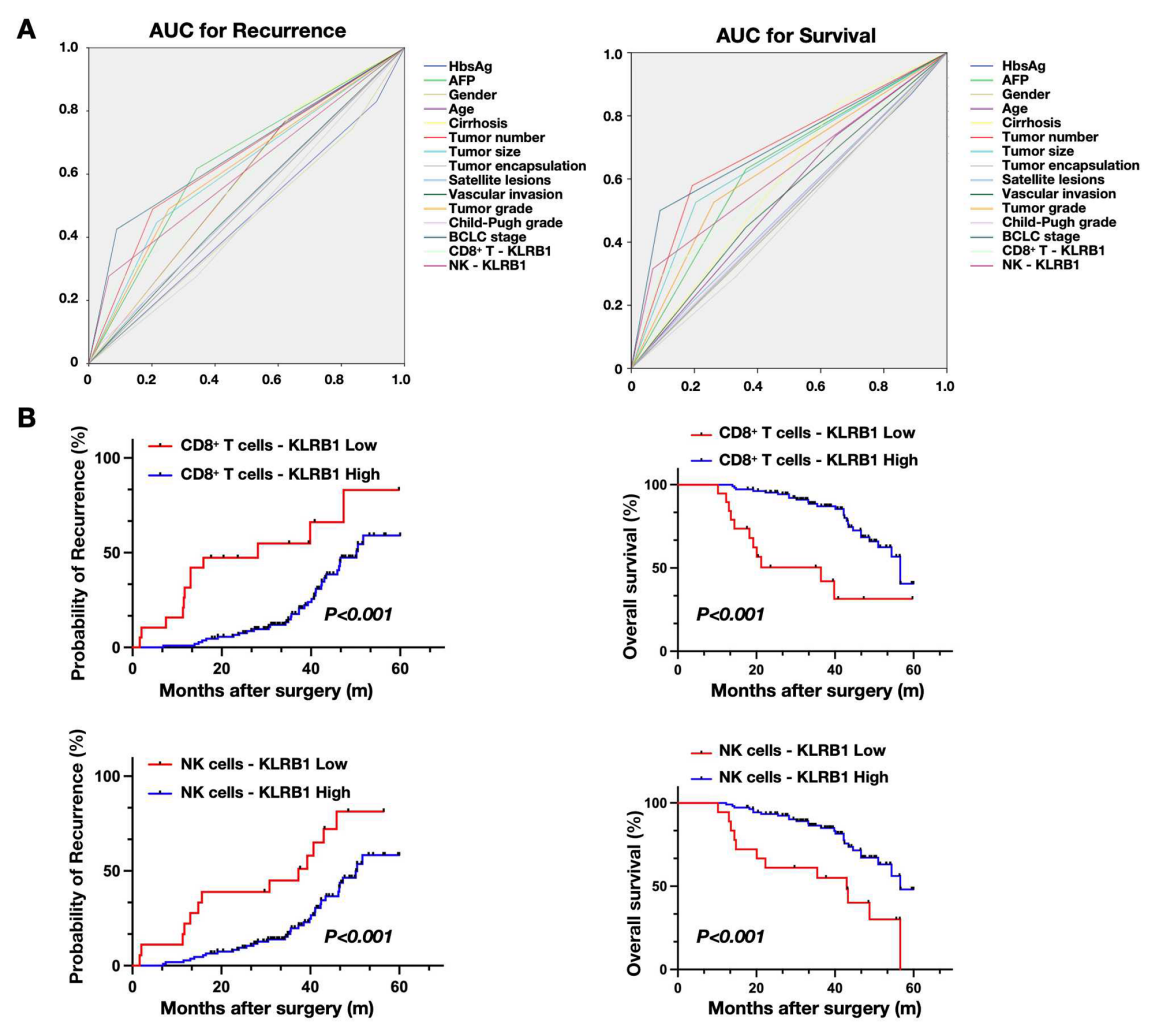
Predictive value of KLRB1 expression on CD8^+^ T cells and NK cells for HCC prognosis. (A) X-tile 3.6.1 software categorized KLRB1 expression on CD8^+^ T cells and NK cells into high and low groups. ROC analysis identified significant predictors of HCC recurrence and mortality, including AFP, tumor number, tumor size, tumor grade, BCLC stage, and KLRB1 expression on CD8^+^ T cells and NK cells. (B) Kaplan-Meier analysis based on KLRB1 expression on CD8^+^ T cells and NK cells showed discriminatory capabilities in prognostic stratification

Based on the aforementioned findings, we hypothesize a strong correlation between the expression levels of KLRB1 on CD8^+^ T cells and NK cells and the prognostic outcomes of HCC patients. To explore this association, we employed X-tile 3.6.1 software (Yale University, New Haven, CT) [21] to categorize the expression of KLRB1 on CD8^+^ T cells and NK cells into high and low expression groups, respectively. Subsequently, KLRB1 expression levels below 17.2% on CD8^+^ T cells and 32.4% on NK cells were classified as low expression, while levels above these thresholds were designated as high expression. We conducted ROC curve analysis to evaluate the predictive efficacy of various clinical parameters in forecasting the prognosis of HCC patients. Our analysis identified AFP(Recurrence, AUC: 0.638, 95%CI: 0.537–0.739; Survival, AUC: 0.634, 95%CI: 0.528–0.740), tumor number(Recurrence, AUC: 0.643, 95%CI: 0.541–0.746; Survival, AUC:0.693, 95%CI: 0.587–0.799), tumor size(Recurrence, AUC: 0.616, 95%CI: 0.512–0.720; Survival, AUC: 0.661, 95%CI: 0.553–0.769), tumor grade(Recurrence, AUC: 0.618, 95%CI: 0.515–0.721; Survival, AUC: 0.632, 95%CI: 0.524–0.741), BCLC stage(Recurrence, AUC: 0.668, 95%CI: 0.565–0.771; Survival, AUC:0.705, 95%CI: 0.596–0.813), as well as the expression of KLRB1 on CD8^+^ T cells(Recurrence, AUC: 0.583, 95%CI: 0.477–0.689; Survival, AUC:0.599, 95%CI: 0.486–0.713) and NK cells(Recurrence, AUC: 0.607, 95%CI: 0.501–0.713; Survival, AUC: 0.624, 95%CI: 0.513–0.737), as significant predictors of recurrence and mortality in HCC patients (Fig. 4A). Moreover, leveraging the differential expression of KLRB1, we stratified HCC patients into two groups and employed Kaplan-Meier analysis to discern whether KLRB1 expression on CD8^+^ T cells and NK cells could effectively differentiate patient prognosis. Notably, both CD8^+^ T cell and NK cell expression of KLRB1 demonstrated discriminatory capabilities in prognostic stratification (Fig. 4B). Finally, we performed COX regression analysis to assess the correlation between clinical parameters of HCC patients and their prognosis survival status. Univariate regression analysis indicated a significant correlation between tumor number, tumor size, tumor stage, BCLC stage, AFP, and the expression of KLRB1 on CD8^+^ T cells and NK cells with patient recurrence and death. Multivariate regression analysis further suggested that tumor number, tumor size, BCLC stage, and the expression of KLRB1 on CD8^+^ T cells and NK cells were independent risk factors for predicting patient prognosis (Tables 3 and 4). These findings underscore the potential of KLRB1 expression levels as prognostic indicators in HCC management.

**Table 3:** Univariate and multivariate Cox proportional hazard analysis of clinical factors associated with recurrence

	Univariate analysis		Multivariate analysis	
	HR(95% CI)	*P* value	HR(95% CI)	*P* value
Gender Male vs. Female	0.626(0.324–1.208)	0.162	NA	NA
Age > 50 vs. ≤ 50	1.410(0.716–2.775)	0.320	NA	NA
Liver cirrhosis Yes vs. No	1.691(0.817-3.500)	0.157	NA	NA
Tumor number Multiple vs. Single	**2.964(1.669–5.263)**	**0.000**	**2.106(1.125–3.941)**	**0.020**
Tumor size > 5 cm vs. ≤ 5 cm	**2.581(1.443–4.619)**	**0.001**	**2.192(1.197–4.013)**	**0.011**
Tumor encapsulation None vs. Complete	0.875(0.461–1.659)	0.682	NA	NA
Satellite lesion Yes vs. NO	1.133(0.559–2.298)	0.728	NA	NA
Vascular invasion Yes vs. NO	1.396(0.776–2.511)	0.265	NA	NA
Tumor grade III-IV vs. I-II	**2.114(1.207–3.810)**	**0.009**	1.372(0.734–2.564)	0.322
Child-Pugh grade B vs. A	0.848(0.262–2.750)	0.784	NA	NA
BCLC stage B + C vs. 0 + A	**3.365(1.882–6.017)**	**0.000**	**2.596(1.307–5.56)**	**0.006**
AFP > 400ng/mL vs. ≤ 400ng/mL	**2.158(1.197-0.3890)**	**0.011**	1.007(0.511-0.985)	0.984
HBsAg Positive vs. Negative	0.738(0.344–1.580)	0.434	NA	NA
CD8^+^ T cells KLRB1 Low vs. High	**0.269(0.139–0.521)**	**0.000**	**0.262(0.125–0.548)**	**0.000**
NK cells KLRB1 Low vs. High	**0.338(0.178–0.643)**	**0.001**	**0.376(0.186–0.759)**	**0.006**

**Table 4:** Univariate and multivariate Cox proportional hazard analysis of clinical factors associated with survival

	Univariate analysis		Multivariate analysis	
	HR(95% CI)	*P* value	HR(95% CI)	*P* value
Gender Male vs. Female	0.850(0.389–1.857)	0.684	NA	NA
Age > 50 vs. ≤ 50	1.318(0.640–2.717)	0.454	NA	NA
Liver cirrhosis Yes vs. No	2.109(0.881–5.045)	0.094	NA	NA
Tumor number Multiple vs. Single	**3.678(1.936–7.025)**	**0.000**	**3.031(1.456–6.308)**	**0.003**
Tumor size > 5 cm vs. ≤ 5 cm	**3.858(1.987–7.490)**	**0.000**	**3.778(1.869–7.636)**	**0.000**
Tumor encapsulation None vs. Complete	0.812(0.401–1.647)	0.565	NA	NA
Satellite lesion Yes vs. NO	1.196(0.545–2.628)	**0.655**	NA	NA
Vascular invasion Yes vs. NO	1.787(0.933–3.422)	0.080	NA	NA
Tumor grade III-IV vs. I-II	**2.442(1.271–4.615)**	**0.007**	0.925(0.457–1.875)	0.829
Child-Pugh grade B vs. A	0.671(0.160–2.807)	0.585	NA	NA
BCLC stage B + C vs. 0 + A	**5.166(2.671–9.992)**	**0.000**	**4.485(2.072–9.709)**	**0.000**
AFP > 400ng/mL vs. ≤ 400ng/mL	**2.192(1.132–4.243)**	**0.020**	0.693(0.312–1.540)	0.368
HBsAg Positive vs. Negative	0.986(0.385–2.530)	0.977	NA	NA
CD8^+^ T cells KLRB1 Low vs. High	**0.220(0.107–0.449)**	**0.000**	**0.199(0.087–0.457)**	**0.000**
NK cells KLRB1 Low vs. High	**0.332(0.167–0.659)**	**0.002**	**0.391(0.184–0.831)**	**0.015**

**Fig. 5:**
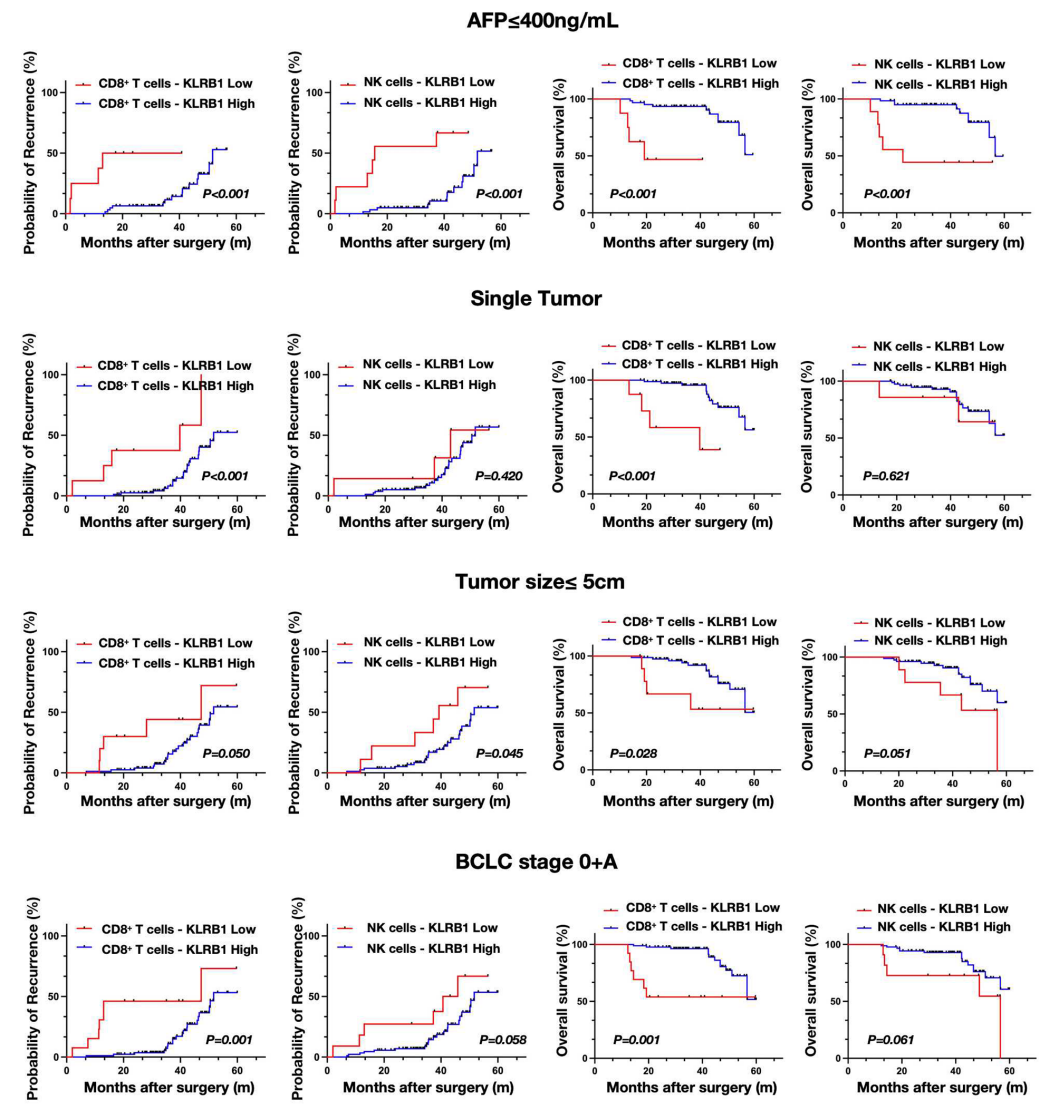
The prognostic value of KLRB1 expression on CD8^+^ T cells and NK cells in the low-risk subgroup of HCC patients

In the subsequent analysis, we examined the prognostic value of KLRB1 expression on CD8 T cells and NK cells within the low-risk subgroup of HCC patients (defined as AFP < 400 ng/mL, single tumor, tumor size < 5 cm and BCLC 0 + A). Our findings revealed that KLRB1 expression on CD8^+^ T cells effectively stratified patients within this low-risk subgroup based on recurrence and survival outcomes. Conversely, the predictive capability of KLRB1 expression on NK cells was found to be insufficient for distinguishing prognosis within the low-risk subgroup of HCC patients, failing to reliably differentiate between those with favorable and unfavorable prognoses (Fig. 5).

## Discussion

KLRB1, initially identified as a highly expressed receptor on rat NK cells, belongs to a class of receptors that, upon activation, can induce NK cell redirection cytotoxicity against FcR + targets and promote the exocytosis of NK cell lytic granules [22]. Subsequently, its homologous gene was discovered in humans [23], with expression primarily observed in human CD56^bright^ CD16^−^ and CD56^dim^ CD16^+^ NK cells, as well as in CD4^+^ and CD8^+^ T cells, NKT cells, and TCRγδ^+^ T cells [24–26]. In this study, through the analysis of the TCGA database and single-cell sequencing data from liver cancer tissues, we observed that KLRB1 is predominantly expressed on NK cells and T cells within liver cancer tissues, with a notably higher expression on NK cells compared to T cells. This finding was subsequently validated by flow cytometry analysis.

Besides, our analysis of the TCGA database has identified KLRB1 as a promising prognostic biomarker for HCC. To further validate this discovery, we collected peripheral blood samples from 126 HCC patients and assessed the expression of KLRB1 on NK cells and T cells. The findings revealed a significant downregulation of KLRB1 expression on both NK cells and T cells compared to healthy controls. Importantly, the expression levels of KLRB1 on CD8 T cells and NK cells effectively stratified the prognostic status of HCC patients. Notably, even within the low-risk subgroup of HCC, the prognostic efficacy of KLRB1 expression on CD8 T cells remained intact. As a hematological marker, KLRB1 offers non-invasive and easily detectable advantages over histopathological findings, rendering it highly suitable for clinical application.

Previous research on KLRB1 has suggested its role in immune regulation across various physiological and pathological conditions of the liver. It has been observed that CD161-positive CD4^+^ and CD8^+^ T cells tend to accumulate in the liver during infections and non-alcoholic fatty liver disease [27]. Patients afflicted with hepatitis B and hepatitis C exhibit heightened CD161 expression on CD8^+^ T cells [28], with studies indicating that CD8^+^ T cells with elevated CD161 levels secrete IL-17 [29]. Notably, this subset of cells also demonstrates elevated expression of the transcription factor RORγt, characteristic of Th17 cells, and are thus referred to as Tc17 cells [30]. The research indicates that Tc17 cells also co-express IL-18Rα. Consequently, these Tc17 cells are regarded as memory stem cells, possessing robust proliferative and differentiation abilities, which enables them to effectively counteract the cytotoxic effects of chemotherapy drugs [31].

Moreover, KLRB1 is emerging as a novel immune checkpoint in tumorigenesis. Its ligand, lectin-like transcript 1 (LLT1), expressed in various tumors, interacts with KLRB1 on immune cells, thereby modulating the immune milieu within tumor microenvironments []. However, the precise role of KLRB1 in tumorigenesis remains elusive and somewhat contradictory. Studies by Li et al. suggested that CD8 T cells exhibiting heightened KLRB1 expression demonstrated augmented cytotoxicity and proliferative capacity in HCC [33]. Conversely, Sun et al. found, through analysis of single-cell sequencing data from recurrent and primary HCC patients, that CD8 + T cells in recurrent tumors exhibited excessive KLRB1 expression and adopted a state of diminished cytotoxicity akin to innate immunity, accompanied by reduced clonal expansion capability [8]. In our study, analysis of KLRB1 using the TCGA database demonstrated a significant association with T cell activation and immune response in hepatocellular carcinoma (HCC). Furthermore, KEGG pathway analysis implicated KLRB1 in the interaction between cytokine receptors and their ligands, suggesting a promotive role in immune activation. Subsequent single-cell sequencing data from liver tumor tissues revealed that NK and T cells with elevated KLRB1 expression lack exhaustion markers and instead exhibit markers indicative of immune cell activation. These findings suggest that KLRB1 acts as a suppressive factor in HCC development.

However, this study has some limitations. Firstly, we did not conduct in vitro cellular experiments on T cells and NK cells exhibiting high KLRB1 expression. Secondly, the sample size of enrolled participants remains relatively small, necessitating further recruitment of a larger cohort. Thirdly, the prevalence of hepatitis B among HCC patients in China may introduce unforeseen effects on the hepatic immune environment. Fourth, in this study, we used X-tile to analyze the cut-off values of KLRB1 in T cells and NK cells. However, we did not include an additional independent cohort of HCC patients to further validate these cut-off values. We plan to enroll a sufficient number of clinical patients to further validate these two cut-off values to ensure their clinical value in prognostic assessment of HCC in future study. Therefore, further research on KLRB1 is warranted.

### Electronic supplementary material

Below is the link to the electronic supplementary material.

Supplementary Material 1

Supplementary Material 2

## Data Availability

The raw data will not be shared. All potential findings based on row data analysis are presented in the manuscript or additional files.

## Abbreviations

AFP: Alpha-fetoprotein
BP: Biological processes
CC: Cellular component
DAVID: Database for Annotation, Visualization, and Integrated Discovery
GO: Gene Ontology
HCC: Hepatocellular carcinoma
IFN-γ: Interferon-gamma
KEGG: Kyoto Encyclopedia of Genes and Genomes
LLT1: Lectin-like transcript 1
MF: Molecular function
NKR-P1A: Natural Killer Cell Receptor 1 A
NK: Natural killer
OS: Overall survival
PBMC: Peripheral blood mononuclear cells
PCA: Principal component analysis
ROC: Receiver operating characteristic curve
TCGA: The Cancer Genome Atlas
TNF-a: Tumor necrosis factor-alpha
Tsne: T-distributed stochastic neighbor embedding
TTR: Time to recurrence
