# Supplementary figures and images for: Deciphering the role of KLRB1: a novel prognostic indicator in hepatocellular carcinoma
Source: BMC Gastroenterol. 2024 Jun 24;24:210. doi: 10.1186/s12876-024-03299-4 (PMC11194965; doi:10.1186/s12876-024-03299-4)

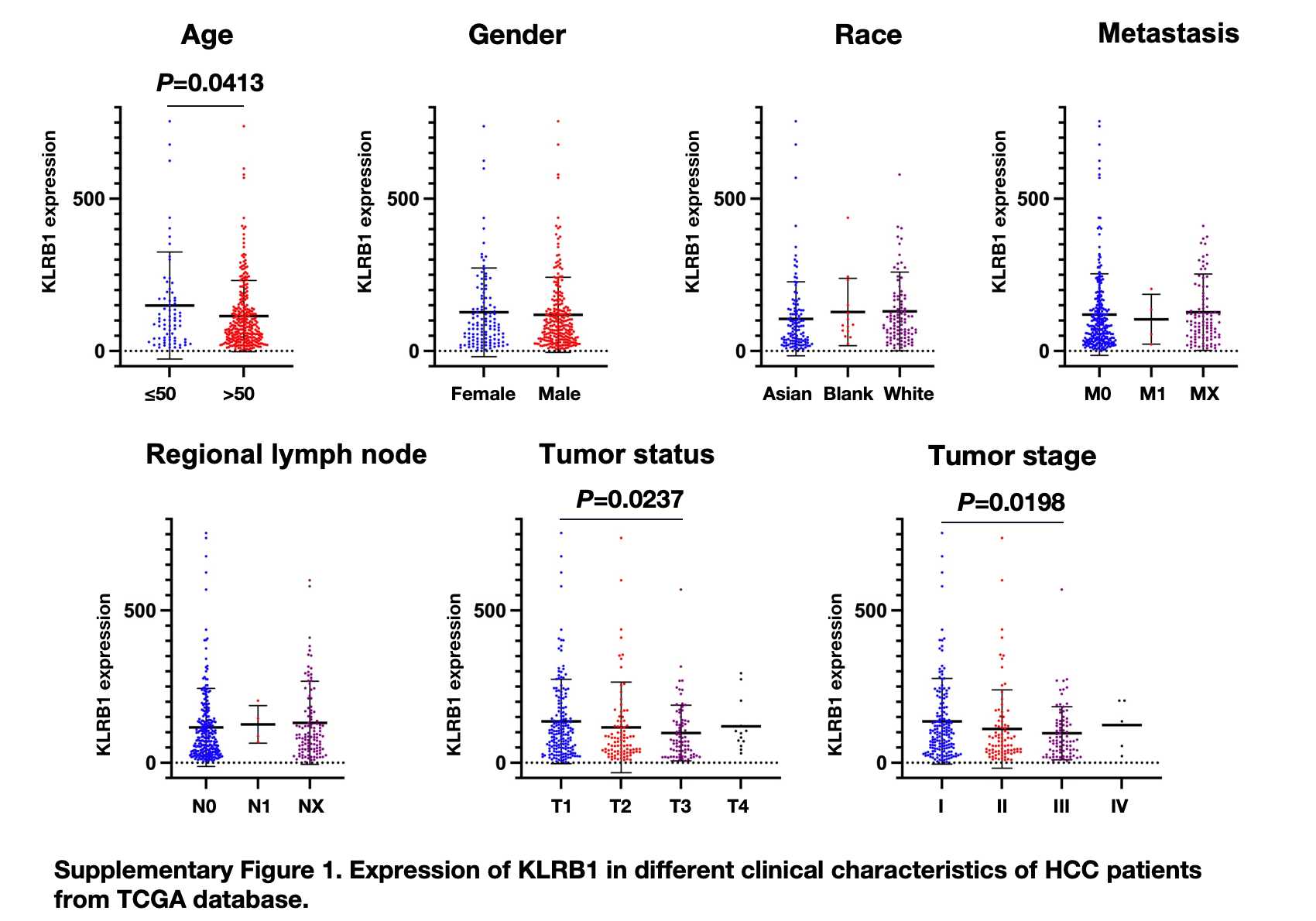

Supplement: Supplementary file 1 — Supplementary Material 1 [file 12876_2024_3299_MOESM1_ESM.jpg]

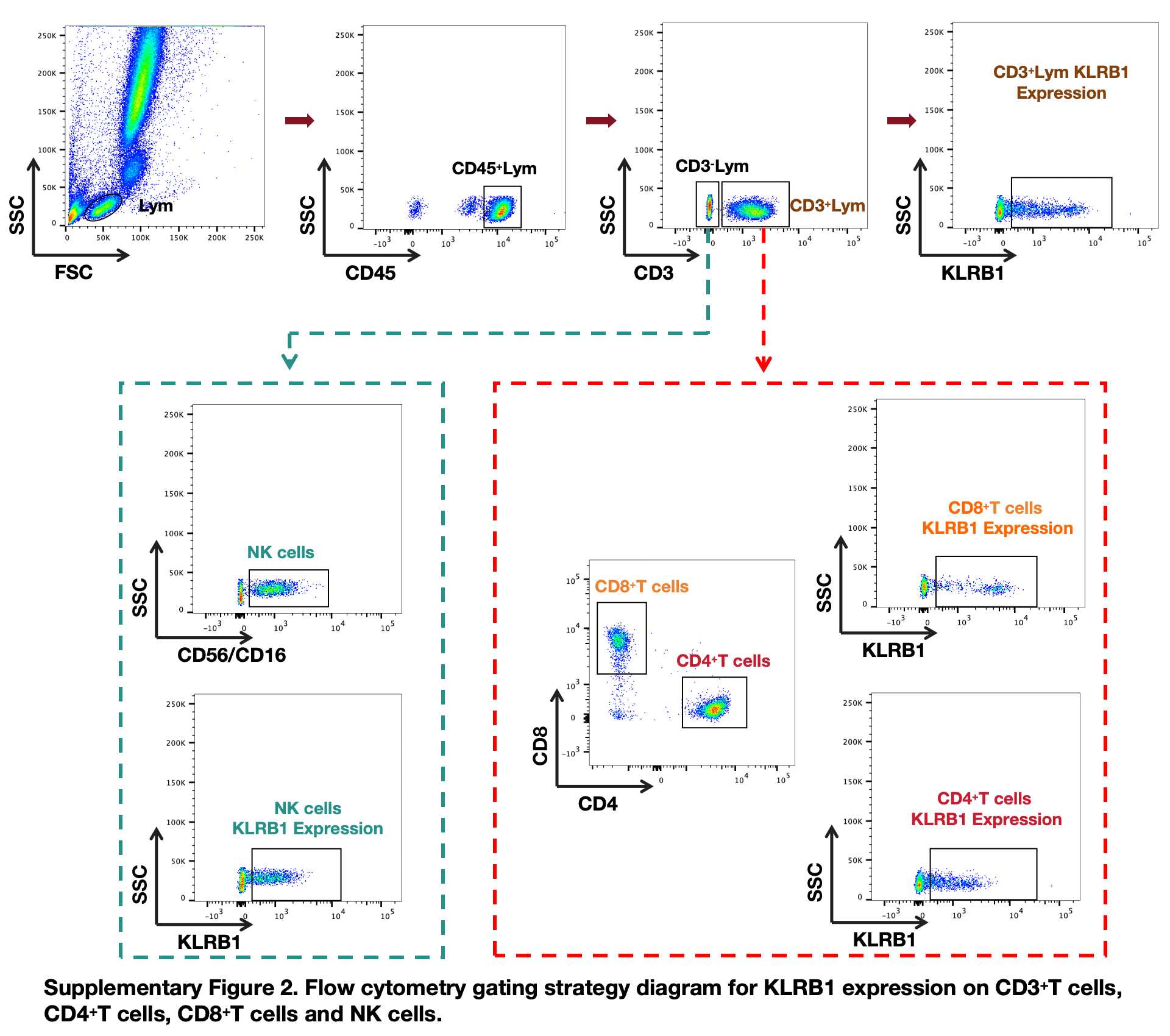

Supplement: Supplementary file 2 — Supplementary Material 2 [file 12876_2024_3299_MOESM2_ESM.jpg]
